# Evolving uses of oral reverse transcriptase inhibitors in the HIV-1 epidemic: from treatment to prevention

**DOI:** 10.1186/1742-4690-10-82

**Published:** 2013-07-31

**Authors:** Ravindra K Gupta, David A M C Van de Vijver, Sheetal Manicklal, Mark A Wainberg

**Affiliations:** 1 Division of Infection and Immunity, University College, 90 Gower St, London WC1E 6BT, UK; 2 Department of Virology, Erasmus Medical Centre, Erasmus University, Rotterdam, the Netherlands; 3 School of Nursing and Public Health, University of KwaZulu-Natal, Durban, South Africa; 4 McGill University AIDS Centre, Jewish General Hospital, Montreal, Canada

## Abstract

The HIV epidemic continues unabated, with no highly effective vaccine and no cure. Each new infection has significant economic, social and human costs and prevention efforts are now as great a priority as global antiretroviral therapy (ART) scale up. Reverse transcriptase inhibitors, the first licensed class of ART, have been at the forefront of treatment and prevention of mother to child transmission over the past two decades. Now, their use in adult prevention is being extensively investigated. We describe two approaches: treatment as prevention (TasP) - the use of combination ART (2NRTI and 1NNRTI) following HIV diagnosis to limit transmission and pre-exposure prophylaxis (PrEP) –the use of single or dual oral agents prior to sexual exposure. Prevention of mother-to-child transmission using NRTI has been highly successful, though does not involve sustained use of NRTI to limit transmission. Despite theoretical and preliminary support for TasP and PrEP, data thus far indicate that adherence, retention in care and late diagnosis are the major barriers to their successful, sustained implementation. Future advances in drug technologies will be needed to overcome the issue of drug adherence, through development of drugs that involve both less frequent dosing as well as reduced toxicity, possibly through specific targeting of infected cells.

## Review

### Introduction

The HIV epidemic has been devastating in its magnitude and devastation [1], despite the availability of effective antiretroviral therapy (ART). There are a number of reasons for this, including lack of access to ART for the majority of infected individuals until relatively recently [2] and low rates of uptake of HIV testing [3]. The global scale up of ART has gathered considerable momentum with an estimated 8 million individuals currently treated, and corresponding reductions in morbidity and mortality have been documented [4,5].

By contrast, in the absence of an effective vaccine and/or cure, transmission has continued largely unabated over the last two decades, particularly in sub Saharan Africa, where 67% [6] of all HIV infections are to be found. Male circumcision has demonstrated around 50% protection in limiting transmission [7], although logistical and ethical barriers may limit its public health impact. Topical microbicides have shown some promise, with vaginal microbicide gel containing tenofovir (TFV) conferring 39% protection in one study [8]. CAPRISA used tenofovir only (as TFV and not as TDF).

Clearly, more effective prevention tools are needed. Prevention of mother to child transmission has proved highly effective when implemented efficiently, and serves as a model for prevention using antiretroviral drugs. Antiretrovirals, the cornerstone of HIV treatment, are now being assessed as tools for limiting transmission in two ways: treatment as prevention (TasP) and pre-exposure prophylaxis (PrEP). The potential for TasP to curb the epidemic is being explored following a report showing that transmission amongst discordant couples was reduced by 96% when the HIV infected partner initiated immediate antiretroviral therapy as compared to delaying treatment until a CD4 <250 cells/mm^3^[9]. A recent study from South Africa has shown a reduction in new HIV infections in a high incidence area following ART scale up with two nucleoside reverse transcriptase inhibitors (NRTI) and one non-nucleoside reverse transcriptase inhibitor (NNRTI) [10]. Modeling studies suggest that universal testing followed by immediate treatment in those who test positive for HIV would lead to a diminution in numbers of new cases and that transmission could eventually be interrupted [11].

In this review, we highlight the achievements of ART in reducing morbidity and mortality, with particular emphasis on reverse transcriptase inhibitors (RTI). We review RTI use in prevention strategies and its anticipated impact on the HIV epidemic. We highlight the potential for drug resistance and the challenge that this presents to implementation of such prevention strategies.

### NRTI and drug resistance

NRTI was the first class of antiretrovirals, with the thymidine analogue zidovudine (ZDV) introduced in 1987 [12]. Subsequent NRTI licensed were the cytosine analogue didanosine (ddI) in 1991, which showed clinical benefit after ZDV exposure [13], as did the thymidine analogue stavudine (d4T) [14]. The cytidine analogue lamivudine (3TC) [15] was licensed in 1995 and was able to restore sensitivity of ZDV resistant virus [16]. Later the guansoine analogue abacavir (ABC) and the nucleotide analogue tenofovir (TFV) were licensed and showed superior outcomes when compared to thymidine analogues [17-19]. Towards the mid 1990s, protease inhibitors (PI) and NNRTI were also introduced, with the combination of 3 or more drugs including a PI or NNRTI along with 2NRTI leading to sustainable suppression in patients with prior AZT exposure [20]. Longer-term benefits from triple drug combination therapy have been observed in different settings [21,22]. The twenty first century has seen introduction of a number of new classes of ART, including integrase strand transfer inhibitors (INSTIs) such as raltegravir and elvitegravir, the CCR5 antagonist maraviroc, and the fusion inhibitor enfurvitide.

Thymidine analogue resistance usually develops in a stepwise fashion, involving mutations (M41L, D67N, K70R, L210W, T215Y or F, K219Q), which increase the drug excision activity of RT [23,24]. These mutations are found at peripheries of the active site and were first described shortly after the era of ZDV monotherapy. Thymidine analogue mutations (TAMs) increase nucleotide and therefore drug excision rates and can compromise activity of newer NRTI. In the case of tenofovir, three TAMs including both M41L and L210W have been associated with virologic failure [25].

Important mutations in the RT active site include L74V, K65R and M184V/I. The latter is selected by and confers resistance to 3TC [26] but increases susceptibility to ZDV, d4T and TFV when K65R is present [27,28]. ABC and ddI select L74V and susceptibility is further reduced by M184V/I [29,30] and TAM [30]. K65R can be selected by d4T, ABC, and ddIsubstantially reducing efficacy of TFV, ddI and ABC [29-33]. Rarer active site mutations such as Q151M can emerge with use of thymidine analogues and confer high-level resistance to all NRTI [31,32].

Connection domain resistance mutations such as G335C/D, N348I, A360I/V, V365I, and A376S have been identified and N348I, in particular, emerges following nevirapine (NVP) exposure [34]. These mutations reduce and/or delay RNAseH activity thereby allowing more time for primer unblocking [35]. The effect is to reduce susceptibility to NRTI, in particular to ZDV [36]. As this region of the HIV-1 genome is not routinely sequenced in drug resistance surveys, there are few data on whether these mutations are transmitted, and thus whether they may compromise treatment as prevention strategies.

Second-generation drugs in various classes with activity in the face of mutations associated with drug resistance to first generation drugs have been developed (for example the NNRTI etravirine, the PIs darunavir and tipranavir, and the INSTI dolutegravir). As ABC and TFV are compromised by mutations selected by older NRTI, these drugs are potentially vulnerable in regard to future use in prevention in areas in which ART scale up has occurred.

### NNRTI and resistance

Two currently licensed NNRTI are efavirenz (EFV) and NVP, and both are highly effective when combined with 2NRTI [37]. Both bind in a hydrophobic pocket and arrest DNA synthesis through allosteric effects. High-level resistance is conferred by various single mutations, includingK103N, Y181C, Y188C/L/H, V106A/M, G180A/S and A98G (reviewed in [38]). HIV-2 and HIV-1 group O are not sensitive to this class of agents due to RT polymorphisms [39,40].

The long plasma half-life of NNRTI predisposes them to development of resistance, particularly when a fixed dose tablet is stopped [41]. This may occur due to sub optimal adherence or drug stock-outs, recently reported to be common across Africa [42]. PI and NNRTI based regimens appear to be largely equivalent in terms of viral suppression rates [43]. However, when virologic failure occurs, NNRTI are associated with higher rates of drug resistance to both the NRTI and NNRTI components of regimens as compared to failure occurring following PI treatment [44]. If transmitted, NNRTI resistance is of particular concern as the odds of viral failure when a major NNRTI mutation pre-exists is approximately two in the first year of therapy, based on data from both Europe [45] and sub-Saharan Africa [46]. There is evidence that drug resistance to NRTI and in particular NNRTI has been rising since ART scale up, with the greatest increases being seen in East African countries [47].

### RTI-based treatment outcomes in the era of HAART

Wide scale availability of ART outside industrialized countries has been largely possible through generic production of fixed dose combinations (FDC) and accreditation/quality control by the World Health Organization. Thymidine analogues have featured in the most widely used regimens along with 3TC/NVP with good outcomes [48,49].

However, NVP interacts with rifampicin-containing tuberculosis therapies and is also associated with potentially serious skin reactions and liver toxicity [50]. Data suggest that EFV may have equivalent or superior efficacy as NVP, and this agent has gradually replaced NVP. EFV itself had been avoided due to concerns regarding congenital birth defects, although it appears from retrospective data that risk is not increased [51].

Rates of viral suppression vary widely across sub Saharan Africa [52], but also among industrialised nations and risk groups [53,54]. Programmatic efficacy, drug supply and adherence have been identified as key indicators of successful viral suppression [42]. Lack of virological monitoring has also been associated with increased prevalence of drug resistance at viral failure, most likely due to later detection of viral rebound [55,56]. Although the relative advantage of viral versus CD4 count monitoring can be debated, there is growing consensus that viral load monitoring is essential [57,58]. With appropriate adherence counseling, viral suppression can be achieved without the need for switch to PI based second line agents, and re-suppression after viral rebound in the absence of treatment switch has been observed [56]. Preservation of second line therapy is highly desirable, as most settings have access to only one or two lines of treatment.

### Treatment as prevention (TasP)

The HPTN052 study showed that immediate treatment of HIV-infected individuals results in a 96% reduction of transmission to an uninfected sexual partner in the context of a discordant couple analysis [9]. A study by Granich et al even predicted that universal HIV testing, followed by immediate treatment in those testing positive could eliminate the epidemic within a decade [11]. It is not likely that Treatment as Prevention (TasP) can prevent all infections as this technique has practical constraints [59].

The first practical constraint of TasP is that adherence to ART initiated at high CD4 counts in the absence of any clinical illness may not reflect that achieved in HPTN052. A number of studies are underway to address this issue.

The second constraint is that immediate treatment is frequently not possible as a substantial proportion of patients are diagnosed relatively late during their infection. For instance, epidemiological studies in resource-rich settings reported that 50% of patients are diagnosed with a CD4 < 350 cells/mm^3^[60-62]. As a consequence, patients with early infections are not identified in a timely manner, whereas these patients acccount for a disproportionally high number of onward transmissions [63,64].

Third, in sub-Saharan Africa there is a problem with retention of patients in clinical care [65-68]. In one setting, only 74% of patients were still in clinical care 12 months after start of antiretroviral treatment [68]. Loss-to-follow-up of patients after start of antiretroviral treatment can have a very detrimental impact. One modelling study even predicted that increasing linkage to care and preventing loss to follow-up provides nearly twice the benefits of universal testing and treatment alone [65]. A final constraint is economic: implementation of TasP requires extensive funding for prolonged periods of time.

Although TasP may not completely eliminate the epidemic, modeling studies suggest that ART could be effective in reducing transmission on a population level [11,69-71]. One modeling study that should be highlighted was done by Eaton et al. who systematically compared twelve independent mathematical models using a set of standardized ART intervention scenarios in South-Africa. One scenario that was standardized was an analysis in which treatment is started in 80% of individuals with a CD4 < 350 cells/μl and in which 85% were still on treatment 3 years later. In this scenario the HIV incidence would be reduced by 35% to 54% 8 years after introduction of ART. Similarly, it was found that the actual scale-up of ART in South-Africa reduced the current incidence by 17% to 32% as compared to when ART would not have been available. A recent South African community based study supports the reduction in transmission that was predicted by mathematical modeling [10].

### Future risk of drug resistance following TasP

There are legitimate concerns regarding these of agents for co-temporaneous treatment for clinical indications and public health guided prevention efforts. Both efforts would potentially be compromised by rising drug resistance [47], especially in resource-limited settings where viral load monitoring, genotypic resistance tests and second line therapy are not widely available. Previous studies showed that availability of such monitoring techniques is associated with a reduction in the emergence of drug resistance [55,59].

Use of boosted PI instead of NNRTI as first line could potentially avoid this problem given that viral failure after modern boosted PI is rarely accompanied by the occurrence of resistance mutations [43,44]. However, boosted PI cannot be given with rifampicin used in the treatment of tuberculosis, posing an important logistical barrier. Cost is a further issue, though boosted PI are now being produced generically and prices are set to fall.

Several modeling papers predicted the future prevalence of transmission of drug resistant HIV-1 in sub-Saharan Africa [57,72]. Phillips et al. predicted that in the absence of viral load monitoring, the prevalence of transmitted drug resistance could increase to 12.4%. Implementation of viral load guided monitoring (based on viral load testing every six months) will result in a prevalence of transmission of drug resistant HIV-1 of about 5-6% [57]. Abbas et al. reported that use of ART initiated at a CD4 < 200 cell/μl in South-Africa (80% coverage) will prevent 20% of HIV infections over ten years but increase drug resistance prevalence to almost 7% [72].

In resource-rich settings, implementation of TasP is not expected to result in a substantial increase of drug resistance. In these settings it is standard practice to perform a genotypic resistance before start of treatment, to estimate the plasma HIV-1 RNA load during treatment [73], and a genotypic test can be performed to detect acquired drug resistance [74], with subsequent selection of a virologically active regimen from a wide selection of available agents. Epidemiological studies have indeed found that the burden of transmitted and acquired drug resistance is declining over time in resource-rich settings [60,75,76].

One mathematical model predicted that over a ten-year time span, TasP can reduce the number of new infections by 34% in Los Angeles County and that at the same time multi-drug resistance can almost double from 4.8% to 9.1% [77]. It should be noted that the size of the problem of transmission of drug resistant HIV is greater in the United States than in other resource-rich settings [75]. It is not known if a similar increase could occur in other resource-rich settings where the prevalence of drug resistance seems to have been reduced during the past years despite of increasing numbers of individuals receiving treatment [76,78].

We do not know how good adherence will be where individuals are treated as part of a TasP strategy. Greater risk taking behavior is possible, with individuals not knowing whether their viral load is suppressed in the absence of point of care viral load testing. Under such a scenario, transmission of drug resistant variants could occur. Although further studies are underway to better evaluate TasP, effects on transmitted drug resistance will be difficult to measure given the long follow-up times that will be required to obtain definitive results.

Due to favourable tolerability/toxicity and efficacy profiles, tenofovir will be the most widely used NRTI in first-line therapy [79,80], and also is likely to be the NRTI of choice in second-line therapy for those who have failed a first line thymidine analogue-based regimen. Of particular concern are data suggesting that TDF (as well as stavudine), when combined with NNRTI where subtype C virus infections predominate, is associated with a high prevalence (around 50%) of the K65R mutation in cases of virological failure [31,81-83]. Subtype C viruses seem more likely to develop K65R based on sequence polymorphisms [38], leading to high- level cross-resistance to all currently approved NRTIs except ZDV [33]. At present, rates of transmitted K65R are low worldwide, and, under trial conditions, cases of transmitted drug resistance involving K65R have not been reported. As TDF becomes more widely available, surveillance for transmitted K65R is important.

### Oral antiretrovirals for use as PrEP

Multiple clinical trials have been undertaken in resource limited settings using TDF/FTC or TDF alone as PrEP for predominantly heterosexual transmission. Clinical trials reported that use of PrEP reduced the risk of infection by 44-75% [84-86]. Two studies, however, did not find any efficacy of PrEP in reducing infections which is most likely attributed to sub-optimal adherence [87,88]. In the PrEP trials, blood drug levels correlated with efficacy, consistent with the notion that non-adherence is a primary reason for failure [84-86].

There is a concern that use of PrEP could result in emergence and transmission of HIV drug resistance [89]. Studies performed before highly active antiretroviral therapy (HAART) became available showed that use of only two NRTI’s could result in the emergence of drug resistance. Therefore, drug resistance could rapidly emerge in individuals that continue using PrEP after they became infected. Because tenofovir and emtricitabine are also recommended in first-line treatment [73,90], use of PrEP could result in the loss of future treatment options [89,91]. Accordingly, the FDA registered the use of TDF/FTC as PrEP under the condition that viral isolates of patients that became infected despite the use of PrEP, are investigated for resistance [92].

Resistance was not common in the trials and was detected in only nine individuals, of who most had an unrecognized acute infection [84-86]. Importantly, regular testing for incident infection was undertaken and prophylaxis was stopped in cases of new infection in PrEP trials. Such testing is unlikely to be achieved in most high prevalence settings and this raises the possibility of selection of drug-resistant viruses following transmission.

Assessing the impact of PrEP on drug resistance will require large-scale epidemiological follow-up studies that are expensive and time-consuming. Mathematical models have therefore been developed which predict the impact of PrEP on the future HIV epidemic and the impact of PrEP on HIV drug resistance [93]. An interesting finding that has been reported across models is that introduction of PrEP in sub-Saharan Africa will result in a reduction of the prevalence of HIV as compared to a situation when only ART is used [72,94-96]. Mathematical models also predict that drug resistance will increase in the coming decade in sub-Saharan Africa as access to ART is expanding. However, resistance due to combination ART, including TDF and FTC, is predicted to far exceed resistance due to PrEP [72,97,98]. One model set in South-Africa found that drug resistance can be limited by using different antiretroviral drugs in treatment than the antiretrovirals that are used for PrEP [72]. They reported that ART and PrEP with overlapping antiretroviral prevents 35% of infections over ten years but increases resistance prevalence to 8.2%. Conversely, using ART and PrEP with non-overlapping aniretrovirals prevents more infections (37%) and reduces resistance prevalence to 7.2% [72]. Additional research on non-overlapping antiretrovirals is therefore required. Classes of antiretrovirals that may be considered as PrEP are CCR5-inhibitors– reported to prevent SIV infections in macaques [99].

Behavioral issues regarding TasP would also apply to wide scale PrEP, where, in the absence of early detection of incident HIV infection, risk behavior combined with intermittent PrEP could contribute to emergence and transmission of drug resistant variants. Multiple studies are underway to better understand risk behavior in the context of PrEP, though there is likely to be considerable heterogeneity of results, based on geographical location, risk group and calendar time [100,101].

### RT inhibitors in prevention of mother to child transmission (PMTCT)

RT inhibitors have a long and distinguished history in PMTCT. A few years after introduction of ZDV, the landmark ACTG 076 study showed that a three component ZDV regimen reduced MTCT from 25% to 8% in the absence of breastfeeding [102]. Transmission risk was associated with the duration of ZDV use, although even shortened perinatal regimens conferred substantial protection [103-105]. Subsequent evidence showed that merely one dose of NVP each to the mother and the infant conferred greater protection than peripartum ZDV alone, and this permitted simplification of MTCT prophylaxis and facilitated wide scale uptake of prophylaxis in resource poor settings [106]. Moreover, when prenatal ZDV was boosted with intrapartum NVP, transmission rates in formula-fed infants fell to as low as 2% [107]. Although combination ART (together with elective Caesarian section and absence of breastfeeding) proved to be highly efficacious in industrialised settings [108,109], cost and feasibility concerns coupled with lack of safety data from developing countries (risk of drug toxicity in mother, adverse pregnancy outcomes, and treatment interruption) limited the wider use of triple ARV prophylaxis. Short course regimens, especially single dose (sd) NVP, have therefore become entrenched as the cornerstone of prophylaxis in poorer parts of the world for women not yet requiring ARV therapy.

Unfortunately, important challenges have ensued. Firstly, the inherently low genetic barrier to resistance of NVP, in addition to its long half-life, increases the likelihood for selection of drug resistant virus. ZDV/3TC for a week following sd NVP has been shown to reduce the emergence of NVP resistance [110]. A high prevalence of NNRTI resistance has been widely documented following exposure to NVP-based prophylaxis, in the case of sd NVP as well as extended daily NVP (ED-NVP), in both mother and infant with deleterious consequences for NVP-based first line HAART [111-116]. Resistance, however, often fades rapidly and progressively in the first year following exposure [112,113,117-119]. As a result, virologic susceptibility may be restored in women initiating NVP based HAART following a minimum of 6 months after intrapartum NVP exposure [115,116,120]. In the case of infants, delayed clearance of NVP resistance may also occur following ED-NVP [121]. Although PI-based HAART is recommended for infants exposed to antiretroviral prophylaxis, drawbacks associated with continuing PIs have evoked strategies to recycle NVP following PI induction [122,123]. However, whether these strategies will be effective following use of ED-NVP remains unknown.

Secondly, in RLS the disastrous impact of unsafe replacement feeding on child survival on the one hand [124] and breast milk transmission on the other have threatened the global success of pre- and perinatal prophylaxis regimens. Emerging data from these settings now provide compelling evidence that covering a recommended six month exclusive breastfeeding period either with maternal combination antiretroviral therapy or, where access to HAART is limited, extended daily infant nevirapine for 14 weeks or 6 months comparably suppresses HIV transmission rates to 1-5% at 6-12 months [130]. Of concern are emerging data that maternal combination therapy during breastfeeding can induce not only NNRTI but concomitant RT resistance in infants who become infected despite prophylaxis - K65R, TAMS and M184V have been reported in breastfed infants [131-133]. The appearance of drug resistant virus in the infant is thought to result from direct breast milk transmission of resistant virus or, more likely, the selection of resistance in the infant as a result of breast milk ARV exposure [134,135]. It is therefore critical that programmes implementing maternal ART have systems in place to optimize adherence and retention in care.

There has been a move from sdNVP to HAART in pregnant and breastfeeding women to limit vertical transmission, with prolonged ART following weaning. However, in a South African study, pregnant women were substantially more likely to be lost to follow-up than non-pregnant women for both pre-ART care and while on ART [136,137], and adherence is a concern with the potential development of drug resistance for both mother and infant [131,132].

## Conclusions

Data suggest that treatment as prevention and PrEP may be used to prevent transmission of HIV, and these could be powerful tools in curbing and reversing the global epidemic. The experience of PMTCT provides evidence for the ability of governments and health systems to target and treat a risk group with single dose ART with the aim of preventing new HIV infections. WHO option B + (life-long ART for pregnant HIV infected women as opposed to limited duration therapy) is now recommended, and future studies will address its success. These data will inform the feasibility of TasP. Adherence has been identified as a key barrier to successful implementation of both strategies, and sustained public health messaging will likely be crucial for success. This is a major research priority.

It should be borne in mind that there are long-term toxicities associated with current and probably future ART [138]. EFV is associated with changes in blood lipid profiles and long-term use would be expected to lead to increased cardiovascular complications [139]. TFV use can lead to reductions in renal function over time, as well as decrease in bone mineral density [140]. Newer RTI are in development and may have improved safety profiles. For example, a prodrug related to TFV termed TAF (tenofovir alafenamide) shows promise as a NRTI, achieving high intracellular but low plasma concentrations and hence reduced renal and bone toxicity with oral doses of less than 10 mg per day, in contrast to the oral TFV dose of 245 mg daily [141].

The field is also in need of new classes for treatment that do not overlap with prevention strategies. Basic science can help identify new targets, for example capsid destabilisation/stabilisation agents, maturation inhibitors and antagonists of viral accessory genes. As new agents would need to be cheap in order to be widely available, dose optimization should be a priority for future clinical trials of new antiretrovirals. Critically, basic science can also assist in the development of long acting agents to address the adherence issues that pervade both therapeutic and preventive HIV strategies. One promising approach is nanoparticle technology that might incorporate RTI as well as other agents [142,143].

## Competing interests

The authors declare that they have no competing interests.

## Authors’ contributions

RG, SM, MWDvdV, MW wrote the manuscript. All authors read and approved the final manuscript.
